# Genetic Diversity and Population Structure of *Leptosphaeria biglobosa* from the Winter Oilseed Rape Region in China

**DOI:** 10.3390/jof9111092

**Published:** 2023-11-09

**Authors:** Kang Zhou, Jing Zhang, Long Yang, Guoqing Li, Mingde Wu

**Affiliations:** 1 The State Key Laboratory of Agricultural Microbiology, Huazhong Agricultural University, Wuhan 430070, China; zhoukang19950601@163.com (K.Z.); zhangjing1007@mail.hzau.edu.cn (J.Z.); yanglong@mail.hzau.edu.cn (L.Y.); guoqingli@mail.hzau.edu.cn (G.L.); 2 Hubei Key Laboratory of Plant Pathology, Huazhong Agricultural University, Wuhan 430070, China

**Keywords:** blackleg, *Leptosphaeria biglobosa*, genetic diversity, population structure, pathogenicity

## Abstract

Phoma stem canker (blackleg), caused by the fungi *Leptosphaeria maculans* (anamorph *Phoma lingam*) and *L. biglobosa*, is one of the most devastating diseases in oilseed rape (*Brassica napus* L.) production worldwide. However, the population structure and genetic variation of *L. biglobosa* populations in China have rarely been investigated. Here, a collection of 214 fungal strains of blackleg from China (including Shaanxi, Jiangxi, Hubei, Jiangsu, Chongqing, Sichuan, Guangxi, Guizhou, Hunan, and Henan) and Europe (France and Ukraine) was identified as *L. biglobosa*. Three simple sequence repeat (SSR) markers were developed to characterize their population structure. The results showed that the Nei’s average gene diversity ranged from 0.6771 for the population from Jiangsu to 0.3009 for the population from Hunan. In addition, most of the genetic variability (96%) occurred within groups and there were only relatively small amounts among groups (4.0%) (*F_ST_* = 0.043, *p* = 0.042 < 0.05). Pairwise population differentiation (*F_ST_*) suggested that significant genetic differentiation was observed between different *L. biglobosa* populations. Bayesian and unweighted average method analysis revealed that these *L. biglobosa* strains were clustered into three branches, and three European strains were similar to those from eastern China. The pathogenicity assay showed that those in Group III were significantly more virulent than those in Group I (t = 2.69, *p* = 0.016). The study also showed that Group III was dominant in Chinese *L. biglobosa* populations, which provides new insights for the further study of population evolution and the management of this pathogen.

## 1. Introduction

Blackleg, caused by *Leptosphaeria maculans*/*L. biglobosa*, is one of the most damaging diseases of *Brassica* spp. [1,2,3,4,5]. The disease has caused significant yield losses in Australia [6], the United Kingdom [7], Canada [8], and France [9]. Blackleg causes global rapeseed losses of up to 160 million US dollars every year [10,11]. Due to their transmission through seeds of various *Brassica* species, *L. maculans* and *L. biglobosa* have a worldwide distribution [12]. The two species frequently coexist on *Brassica* crops in Europe, but the relative proportions of their populations in different countries are variable [13]. *L. maculans* is more aggressive and usually causes severe epidemics and substantial yield losses [7,14,15], and is associated with damaging stem base cankers [16]. There is also evidence that *L. maculans* is slowly invading areas where *L. biglobosa* was previously present [17]. For example, *L. biglobosa* was prevalent in Poland until the mid-1990s [18]. By 2002, *L. maculans* was widespread in western Poland, while only *L. biglobosa* was found in eastern Poland [19]. China has the largest planting area of *Brassica* crops in the world. However, only *L. biglobosa* has been reported to date [20]. Blackleg of oilseed rape (*L. biglobosa*) was first reported in the Anhui, Hubei, and Guizhou provinces of China [17]. Since then, *L. biglobosa* has been reported in all major oilseed-rape-producing regions in China [21]. Although *L. maculans* has not been detected in these oilseed-rape-producing regions, there is still a great risk of its introduction into China, which would cause great economic losses [3,20,22].

In recent years, the understanding of the genetic and geographic variation in *L*. *maculans*/*L*. *biglobosa* populations has significantly improved [23,24]. A variety of molecular techniques are now available for the rapid and dependable identification of genetic variations in their populations. For example, minisatellite markers were developed to describe the genetic diversity of *L. maculans* in France [25,26]. Random amplified polymorphic or microsatellite was used to identify the high genetic variability of *L. maculans* isolates [27,28,29]. In China, amplified fragment length polymorphism (AFLP) or inter-simple sequence repeat (ISSR) markers were also used to analyze the genetic diversity of *L. biglobosa*. The results showed that all isolates of blackleg from China were identified as *L. biglobosa* ‘brassica’, and that the *L. biglobosa* populations in China were more similar to those in Europe [30,31]. However, the population structure of *L. biglobosa* in China remains unclear.

Microsatellite markers are also known as simple sequence repeats (SSR), referring to tandem repeats with 1–7 nucleotides [32,33,34], and are often used for genetic diversity research [35]. These are widely distributed in the genome with high genetic variation, and are generally more reliable than other genetic markers (AFLP or randomly amplified polymorphic DNA, RAPD) [36]. To date, no study on *L. biglobosa* population diversity based on the SSR marker has been reported. In addition, previous studies showed that the virulence of some fungal pathogens was also associated with their genetic clusters [37,38]. Therefore, the objectives of this study were (i) to develop an SSR-based method for the population study of *L. biglobosa* ‘brassica’; (ii) to characterize the genetic diversity and population structure of *L. biglobosa* ‘brassica’ in the winter oilseed region of China; and (iii) to test the virulence of different genetic clusters.

## 2. Materials and Methods

### 2.1. Fungal Strains and DNA Extraction

**Figure 1:**
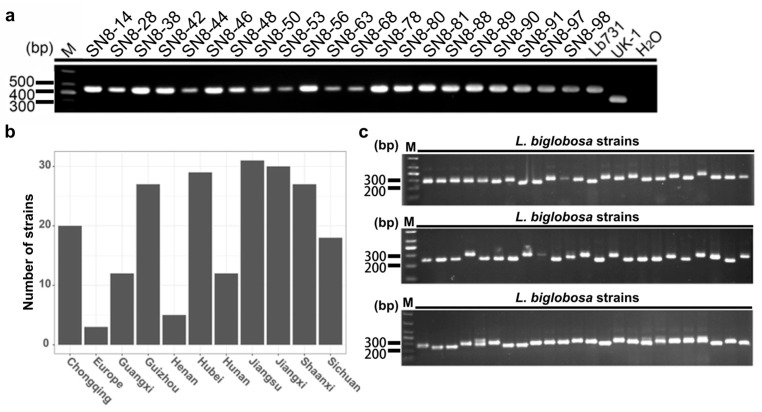
Molecular characterization of *Leptosphaeria biglobosa* strains from different oilseed rape growing regions in China. (**a**) Electrophoresis analysis of 21 *L. biglobosa* strains based on species-specific primers (LmacF, LmacR, and LbigF) in 1.5% agarose gel, and strains Lb731 and UK-1 served as positive controls of *L. biglobosa* and *L*. *maculans*, respectively. (**b**) The numbers and geographic origin of *L. biglobosa* strains used for SSR analysis. (**c**) Electrophoresis analysis of 24 *L. biglobosa* strains based on ssr-14 primers in 2% agarose gel.

A total of 211 strains of *L. biglobosa* ‘brassica’ were obtained from diseased oilseed rape showing symptoms of blackleg in 10 provinces (Shaanxi, Jiangxi, Hubei, Jiangsu, Chongqing, Sichuan, Guangxi, Guizhou, Hunan, and Henan) in China in 2018 (Figure 1b) using the method described in our previous study [20]. Several 2 mm × 2 mm pieces of diseased tissue were clipped from the disease samples with scissors for surface disinfection. The tissue was first disinfected with 75% (*v*/*v*) alcohol for 1 min, then treated with 5% (*v*/*v*) NaClO for 2 min, and finally rinsed with sterile water three times. After the disinfection, the tissues were placed on a potato dextrose agar (PDA) plate containing 0.5% (*v*/*s*) lactic acid with sterilized tweezers, 4–5 tissues per plate, and the surface water was air-dried on a clean bench. The plates were then placed at 23 °C and cultured for 2–3 days without light. After the appearance of mycelial growth, a mycelial plug from the colony margin was transferred onto a new PDA plate. Each plate was inoculated with one mycelial plug, and purified strains were obtained after consecutive sub-culturing three times. For simplification, in the following text, we only mention *L*. *biglobosa* instead of *L. biglobosa* ‘brassica’. In addition, two *L. biglobosa* strains obtained from the imported seeds from France and Ukraine were also included. All *L. biglobosa* strains were grown on potato dextrose agar (PDA) and stored at −80 °C in 20% glycerol.

For DNA extraction, the mycelial plugs of each strain were inoculated on a PDA plate (five plugs per plate) covered with cellophane for four days, and the mycelium was then collected for DNA extraction. The genomic DNA of each *L*. *biglobosa* strain was extracted using the cetyltrime-thylammonium bromide (CTAB) method [39]. Mycelium weighing 0.2 g was quickly ground into powder in liquid nitrogen and transferred into a 2 mL EP tube containing 800 μL CTAB buffer. After being evenly mixed, the mixture was heated at 65 °C in a water bath for 0.5 h, then 400 μL of phenol and 400 μL of chloroform–isoamyl alcohol (24:1) were added and evenly mixed. The mixture was centrifuged at 12,000 rpm for 10 min, and the supernatant was taken. This process was repeated once. Then, the supernatant was mixed with 800 μL of chloroform–isoamyl alcohol and centrifuged at 12,000 rpm for 10 min. The final supernatant was taken, and two times the volume of anhydrous ethanol was added to the supernatant. After being evenly mixed, the mixture was treated at −20 °C for 2 h, then centrifuged at 12,000 rpm for 10 min. The supernatant was removed, and the DNA precipitation was then washed with 100 μL of 75% ethanol twice. After being air-dried, the genomic DNA was then dissolved in 50 μL of ddH_2_O and stored at −20 °C.

### 2.2. Fungal Species Determination

Species-specific multiplex polymerase chain reaction (PCR) was used to determine the identity of the fungal strains with the species-specific primers LmacF (*L. maculans*) 5′-CTTGCCCACCAATTGGATCCCCTA-3′, LbigF (*L. biglobosa*) 5′-ATCAGGGGATTGGTGTCAGCAGTTGA-3′, and LmacR 5′-GCAAAATGTGCTGCGCTCCAGG-3′ [40]. A PCR product of 444 bp indicates the presence of *L. biglobosa* and a 334 bp fragment indicates the presence of *L. maculans*.

### 2.3. Design of SSR Primers

The genomic sequence of *L. biglobosa* strain G12–14 (National Center for Biotechnology Information accession number PRJEB24467) was used for the SSR search. All scaffolds were screened for SSR motifs using SSRHunter v1.3, and primer pairs were designed using Primer5 [41]. Twenty primer pairs containing an SSR repeat length (36 to 84 bp) were selected, and they were added with fluorescent labeling FAM to the 5’-ends.

### 2.4. Determination of Genetic Diversity of L. biglobosa Strains

PCR was performed at a 50 °C to 56 °C annealing temperature using three SSR primer pairs (ssr-14, ssr-17 and ssr-53, Table 1). Each PCR was conducted in a 25 μL mixture containing 12.5 μL premix (Taq DNA polymerase 0.625 U/12.5 μL premix (TaKaRa, Dalian, China)), 10 pmol/μL primers each at 1 μL, 1 μL of 20 ng/μL fungal genomic DNA, and 9.5 μL ddH_2_O. The thermocycler (Bio-rad S1000^TM^ Thermal Cycler, USA) program for PCR was set for 3 min at 94 °C, followed by 32 cycles of 15 s at 94 °C, 30 s annealing at 52 °C, 10 s extension at 72 °C, and a final extension of 5 min at 72 °C. PCR products were visualized in 2% agarose gels (Biowest, Hong Kong, China) with GelRed (Saibeike, Beijing, China). The amplified products were sequenced and used for fluorescent capillary electrophoresis detection using Tianyi Huayu Gene Technology (Wuhan, China) to determine the sequence and the size of the target fragment [42], respectively.

Data of the amplified fragment length were loaded into the software PowerMarker v3.25 [43] for the calculation of the number and frequency of alleles. GenAlEx v6.5 [44] software was used to calculate the number of genotypes in different microsatellite loci and geographic populations, the number of observed alleles (*Na*), the number of effective alleles (*Ne*), Shannon information index (*I*), Nei’s (1973) Gene Diversity Index (*H*), and Nei’s genetic distance. It was also used for the molecular analysis of variance.

The genetic frequency of each locus was calculated for different strains through Powermarker v3.25 [43]. A tree of the unweighted average method was constructed for different geographical populations based on Nei’s genetic distance. According to the band sizes of different microsatellite amplification products of 214 *L. biglobosa* strains, all strains were clustered into different clusters using Structure v2.3.4 [45].

### 2.5. Pathogenicity Assays

Nine strains originating from different geographical regions were selected for each group, tested on the cotyledons of oilseed rape (*B. napus* L.) cultivar “Zhongshuang No. 9” for the pathogenicity and inoculation of three oilseed rape seedlings per strain according to the method previously described [46]. Each cotyledon was wounded and inoculated with a 10 μL inoculum droplet (10^7^ conidia/mL). Inoculated plants were placed in a greenhouse at 20 °C and 100% RH under 12 h light/12 h dark for 7 days.

## 3. Results

### 3.1. Species Identification and SSR Primer Design

**Figure 2:**
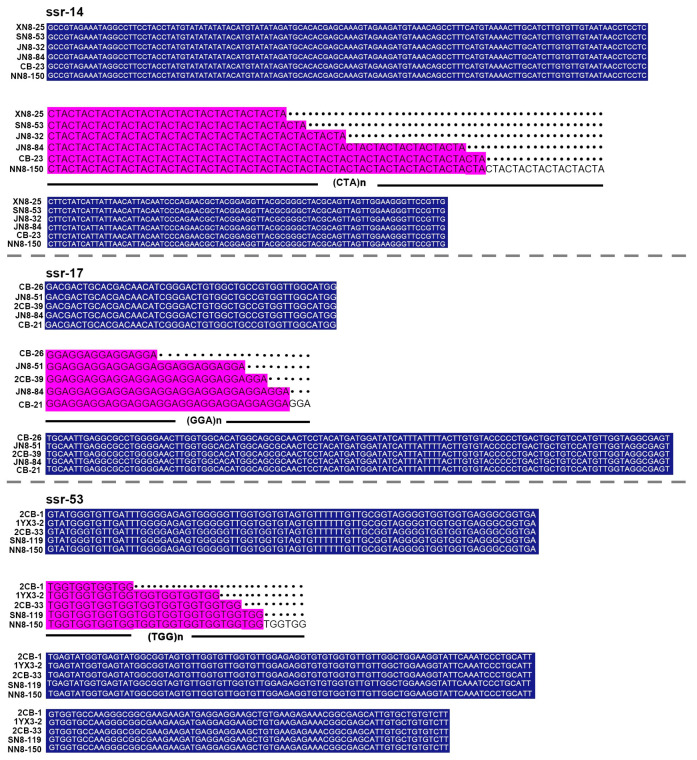
Multiple sequence alignments of the sequences of PCR products based on ssr-14, ssr-17, and ssr-53 primers in partial strains of *L*. *biglobosa*. Sequences in dark blue regions indicate the upstream and downstream of the SSR fragments, and sequences in purple regions indicate the repeat sequences.

The species-specific PCR detection showed that all tested strains were *L. biglobosa* ‘brassica’, as they generated a band 444 bp in size (Figure 1a). A total of 20 pairs of SSR primers were designed using SSRHunter software v1.3, and their PCR products were screened using 2% agarose gel electrophoresis (Figure 1c). Although different target bands were detected by these primers, the products of only three SSR primer pairs (ssr-14, ssr-17, and ssr-53) showed high polymorphism (Table 1), and these three primer pairs were selected for further analysis. The amplified fragments with different sizes were also cloned and sequenced. The results showed that the products of ssr-14 showed the highest polymorphism with 15 kinds of trinucleotide repeat sequences (CTA)_n_ (n = 12, 13, 14, 15, 16, 19, 21, 22, 23, 26, 27, 28, 29, 30, 32). In addition, the products of ssr-17 and ssr-53 had five and six kinds of trinucleotide repeat sequences, respectively. The products of ssr-17 had the trinucleotide repeat sequences (GGA)_n_ (n = 5, 9, 10, 11, 12), while the products of ssr-53 had the trinucleotide repeat sequences (TGG)_n_ (n = 4, 8, 9, 10, 12, 13) (Figure 2).

### 3.2. Genetic Diversity and Population Structure Analysis

**Figure 3:**
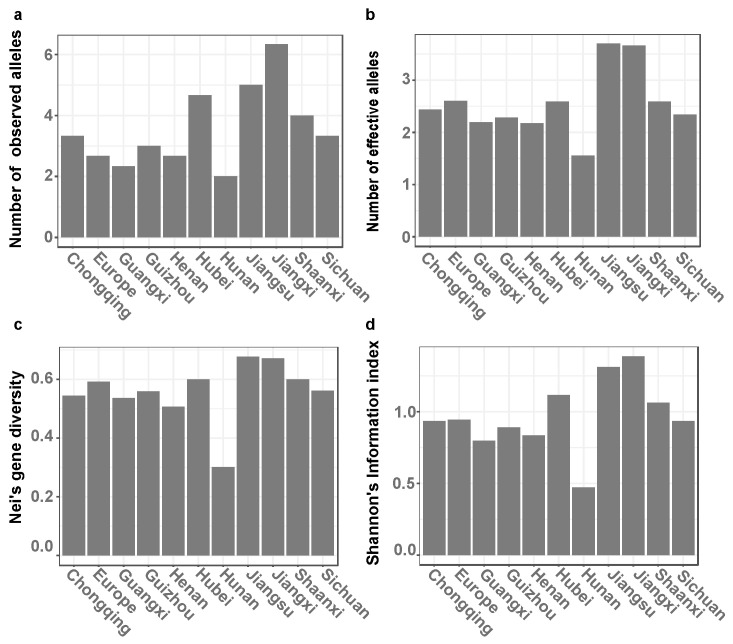
Genetic diversity analysis ((**a**–**d**), the number of observed alleles, the number of effective alleles, Nei’s gene diversity, and Shannon’s Information index) of *Leptosphaeria biglobosa* populations from winter oilseed rape regions in China and Europe.

**Figure 4:**
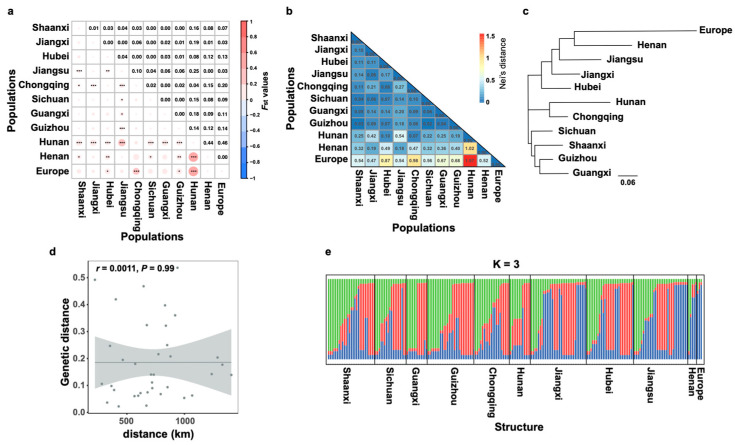
Population structure of *Leptosphaeria biglobosa* from Chinese winter oilseed rape region. (**a**) The pairwise population differentiation (*F_ST_*) among 11 collection locations of *L. biglobosa* based on the SSR datasets. Above the diagonal: *F_ST_* values; below diagonal: *, ** and *** indicates that there is a significant difference at *p* < 0.05, 0.01, and 0.001, respectively. (**b**) The Nei’s genetic distance among 11 collection locations of *L. biglobosa*. (**c**) The clustering relationship of *L. biglobosa* populations of different regions in China based on the genetic distance. (**d**) The correlation analysis between spatial distance and genetic distance of *L. biglobosa* populations in China. (**e**) The structure analysis of *L. biglobosa* populations in China and Europe at K = 3.

There were 5 to 15 alleles observed per locus, and 26 alleles in total were scored with an average of 8.6667 alleles per locus. Among the populations, the average number of effective alleles ranged from 1.5581 to 3.6980. The Nei’s gene diversity and Shannon’s information index varied from 0.3009 to 0.6771 and from 0.4715 to 1.3869, respectively. The gene diversity of the *L. biglobosa* population from Europe (*Na* = 2.6667; *Ne* = 2.6000; *H* = 0.5926; *I* = 0.9446) was in the region of the *L. biglobosa* populations from the winter oilseed rape in China (Figure 3a–d). Gene flow analysis (*Nm* = 5.639) indicated the presence of genetic exchange or migration between different national regions (Table 2). Additionally, the pairwise genetic differentiation values (*F_ST_*) between the 11 populations ranged from 0 to 0.46 based on the SSR analysis. The highest F_ST_ values were observed in pairwise comparisons between populations from Hunan and Europe (Figure 4a). In addition, the AMOVA analysis also showed that 4% (*p* = 0.042 < 0.05) of variations between populations could be attributed to differences among populations, and 96% of the variations were from genetic variation within populations (Table 2). Overall, there were small differences among *L. biglobosa* populations from most regions of winter oilseed rape (Figure 4b). A cluster analysis of populations from different geographical regions showed that they could be divided into three groups, the near-west (Sichuan, Shaanxi, Guizhou, and Guangxi), the near-central (Chongqing, Hunan), and the near-east (Jiangsu, Jiangxi, Hubei, Henan, and Europe) (Figure 4c). Simultaneously, no significant correlation was observed between the genetic distance and the spatial distance of different *L. biglobosa* populations (Figure 4d).

The assignment of population structure was carried out via Bayesian clustering implemented using the Structure software v2.3.4. Posterior probabilities calculated by Structure showed that the best population structure occurred at K = 3. An admixture model was used, which assumes that the individual has a fraction of their genome inherited from a population and other fractions from other populations. The pedigree composition of *L. biglobosa* strains from Europe is more close to the near-east (Henan, Jiangsu, Jiangxi, and Hubei) (Figure 4e).

**Figure 5:**
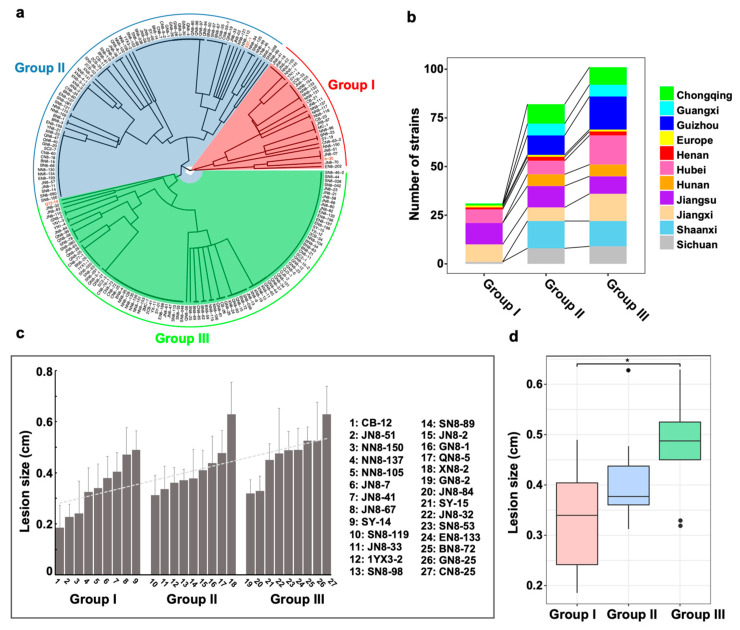
Pathogenicity of strains in three different genetic lineages. (**a**) The cluster of 214 *L. biglobosa* strains based on the non-weighted average method, and the three European strains are labeled in red. (**b**) The number of strains in three clusters. (**c**) The pathogenicity of 27 strains from three groups at 7 dpi. (**d**) The average pathogenicity of populations from three clusters, and “*” indicates that there is a significant difference (*p* < 0.05).

Based on UPGMA cluster analysis, all the *L. biglobosa* strains can be classified into three groups (Figure 5a). Group I contains 31 strains from Jiangxi, Jiangsu, Henan, Hubei, Chongqing, Sichuan, and France; Group II contains 83 strains from 11 different regions. Group III has a dominating ratio of 100 strains from all locations (Figure 5b).

### 3.3. Pathogenicity of the L. biglobosa Strains in Different Groups

The pathogenicity assay showed that all selected *L. biglobosa* strains were able to cause typical leaf lesions on oilseed rape (cultivar “Zhongshuang No. 9”) cotyledons. After 7 dpi, the average lesion diameters of nine strains in Group I ranged from 1.9 to 4.9 mm, while they ranged from 3.1 to 6.3 mm in Group II but were not significantly different from those in Group I (Figure 5c,d). The average lesion diameter of the nine strains in Group III was from 3.2 to 6.3 mm (Figure 5c), which was significantly higher than that of Group I (*p* < 0.05) (Figure 5d).

## 4. Discussion

China is one of the largest oilseed rape production countries in the world. In 2013, the area of oilseed rape in China reached more than 7.5 million hectares, accounting for about 40% of the national oil crop sown [47]. Blackleg is resurging as a major problem for global oilseed rape production. In the present study, all strains were collected from diseased plants showing stem cankers in the winter oilseed rape growing region in China and were confirmed as *L. biglobosa*. This is consistent with the results reported by previous studies, which indicated that 60 and 84 strains collected from oilseed rape showing blackleg symptoms, respectively, were identified as *L. biglobosa*, and no *L. maculans* was detected [30,31]. However, the invasion of *L. maculans* into China is still a huge risk for Chinese oilseed rape production.

Compared with RAPD-PCR and ISSR-PCR, using SSR to access fungal population diversity is more stable and reproducible [48]. In this study, SSR molecular markers based on the *L. biglobosa* genome were established, and three SSR molecular markers with high stability, accuracy, and repeatability were obtained. This is the first report of analysis of the *L. biglobosa* population structure based on SSR markers, and the *L. biglobosa* population used for the study has greatly increased as well.

The genetic diversity and population structure of *L. biglobosa* in China have been rarely reported. A previous study based on the AFLP method showed that the genetic diversity of the Chinese *L. biglobosa* population (33 strains) was lower than the Canadian population, which was genetically more similar to European *L. biglobosa* populations [30]. This result was similar to another study based on ISSR-PCR [31], which showed that the Chinese *L. biglobosa* population was more similar to that from the United Kingdom. Similarly, the present study showed that three European strains were more closely related to the *L. biglobosa* population from Henan, indicating a close relationship between *L. biglobosa* populations from China and Europe. However, the accuracy of genetic diversity estimation is related to the number of strains. If the number is less than three, the genetic diversity in some regions cannot be revealed [47,49]. Therefore, more strains form Europe are required for further accurate evaluation of the genetic distance between China and Europe. In addition, the genetic diversity of *L. biglobosa* populations in Jiangsu was higher than that in Europe or other regions of China. Therefore, it is possible that *L. biglobosa* originated in the east and then spread to the west in the Chinese winter oilseed rape growing region.

Based on the genetic distance between different *L. biglobosa* populations, the *L. biglobosa* populations in the winter oilseed rape region of China could be divided into three large geographic groups: the near-west (Sichuan, Shaanxi, Guizhou, and Guangxi), the near-central (Chongqing and Hunan), and the near-east (Jiangsu, Jiangxi, Hubei, and Henan) (Figure 4c). However, the clustering based on the SSR dataset demonstrated that the *L. biglobosa* populations from China could be divided into three groups, and the genetic variability was not related to their geographic origin (Figure 4d). We suppose that this may be due to the long-distance transportation of rapeseed, which facilitates significant population exchanges of the pathogen *L. biglobosa* between distant regions. Like most fungal introductions, an unintended consequence of human-mediated movement and trade seems to be a reason that the *L. biglobosa* populations had larger gene flow (*Nm* = 5.639) and small genetic differentiation (*F_ST_* = 0.043). The long-term and long-distance transmission of *L. biglobosa* could be achieved by the introduction of seeds [50]. Another explanation is that *L. biglobosa* may spread between different regions through the infection of wild plants [51], as it has a relatively wide host range. Recent studies have also shown that *L. biglobosa*, having a heterothallic mating system, may reproduce sexually, which could also increase the gene flow among different populations [52].

Blackleg was first reported on canola (*Brassica napus*) in North Dakota in 1991, and only two pathogenicity groups, PG-1 (reclassified as *L. biglobosa*) and PG-2 (*L. maculan*), were discovered, with PG-2 being the most abundant group [53]. In 2003, Bradley et al. [54] discovered two new pathogenicity groups (PG-3 and PG-4) in normal blackleg cankers, which accounted for fewer than 5% and 1% of all tested isolates, respectively. However, in 2009, only 3% of the tested isolates obtained from North Dakota belonged to PG-2, while PG-4 and PG-3 strains accounted for 51 and 25%, respectively [55]. Therefore, the population structure of plant pathogens may change dramatically over time. In the present study, three *L. biglobosa* groups generated by the unweighted average method or Bayesian cluster analysis showed differences in virulence, of which Group III showed the highest virulence (t = 2.69, *p* = 0.016 < 0.05), while Group I showed the weakest. Hence, different virulence groups might also exist in Chinese *L. biglobosa* populations. In addition, Group III had the largest population, accounting for 46.7% of the total strains, implying it might be the dominant type in the Chinese natural population of *L. biglobosa*.

## 5. Conclusions

In this study, the identity of the fungal pathogen causing blackleg in the winter oilseed rape region of China was determined to be *L*. *biglobosa* without *L*. *maculans* by using PCR identification. The diversity of *L*. *biglobosa* from Chinese winter oilseed rape based on SSR markers was determined, and the genetic diversity analysis (the number of observed alleles, the number of effective alleles, Nei’s gene diversity, and Shannon’s Information index) and Nei’s genetic distance of *L*. *biglobosa* populations revealed that there are genetic differences between *L*. *biglobosa* populations from different regions. Pairwise population differentiation (*F_ST_*) also suggested that these differentiations were significant (*p* = 0.042 < 0.05). Distance–decay analysis showed that the genetic distances of *L. biglobosa* populations were not closely related to spatial distance (*p* > 0.5). Three groups clustered by UPGMA, having differences in virulence, were identified, and Group III showed the highest virulence (t = 2.69, *p* = 0.016 < 0.05). Further research to monitor potential migration among populations of the pathogen at different locations would advance our understanding of the spread of the pathogen globally.

## Figures and Tables

**Table 1:** Primer sequences, repeat motifs, size ranges, and number of alleles of 20 paired SSR markers.

Locus Name	Repeat Motif	Primer Sequence (5′-3′)	Size (bp)	Allele No.
ssr-1	(CCT)_n_	CTCTCTACCTCCTCCCCCTC	278	1
		CGCAGCGTTTAGGCTTTATC		
ssr-7	(AGC)_n_	GGCGTTTGGATGTGAGAAGT	178	1
		CGTTCACTTTGGGGACAAGT		
ssr-8	(GATGGA)_n_	CTGGGCCTAGAGCAAGAACA	242	1
		TAGAATTCCATGTCCAGCCC		
ssr-12	(CTT)_n_	GCAAGACAGACGGACAGACA	270	1
		GCGGGGTGAGAATTCTTGTA		
ssr-14	(CTA)_n_	GCCGTAGAAATAGGCCTTCC	241–301	15
		CAACGGAACCCTTCCAACTA		
ssr-16	(GGC)_n_	GATTGGCGAGCCTAGAAGTG	262	1
		AGTATTGAATGCAGACCCGC		
ssr-17	(GGA)_n_	GACGACTGCACGACAACATC	178–199	5
		ACTCGCCTACCAACATGGAC		
ssr-23	(GAGT)_n_	CGCAAGTATGAATGCGAAAA	155	1
		CCTTGCAAAGTCGGTCAAGT		
ssr-24	(AGC)_n_	TCGCTGCTGAACAAGTGAGT	245	1
		TAGGCGAATACACGGGAAAC		
ssr-26	(CAG)_n_	GGGCAAAACCAGAGAAGACA	224	1
		GACGACGGCGAGAGACTTAC		
ssr-31	(TTG)_n_	AGGAGTGGGAGAGGCATTTT	274	1
		TGTAAGTCGACTGCGTTTGG		
ssr-34	(TGGA)_n_	TTTGGTGTGATGTCAGGGAG	202	1
		TGTGACAATCTTGCCAAAGC		
ssr-36	(TCG)_n_	GCAACTTGTCGATTCCGACT	271	1
		ACCCAAGTCCTCTGCAGCTA		
ssr-43	(ACCT)_n_	GCTTTGCGAGGTCAAATGTT	204	1
		GCTAGTCAGGACGGGGTAGG		
ssr-44	(CGG)_n_	TTTGAGCTCGACGACATGAG	176	1
		TTTTGGTCTGCCAGCTTCTT		
ssr-45	(GGC)_n_	GGTCTCGTGTGCAATTGATG	268	1
		GCGCAGGCGAGTACATAGTT		
ssr-47	(CTG)_n_	ATCGTCGTCTTGAGCTGGTT	200	1
		AAAGTCTGCATGTCCATCCC		
ssr-52	(GCC)_n_	GAAGTGTCTGCGCCATGTTA	246	1
		ACCTCCGACACCACCTCTC		
ssr-53	(TGG)_n_	GTATGGGTGTTGATTTGGGG	254–281	6
		AAGACACAGCACAATGCTCG		
ssr-56	(TGA)_n_	ACCGTCAGAGAACATACCCG	234	1
		GAGCTTGATCTCCGCTGACT		

**Table 2:** Analysis of molecular variance (AMOVA) between and within populations of *Leptosphaeria biglobosa* determined by three simple sequence repeat (SSR) markers.

Source	df	Sum of Squares	Mean Squares	Estimated Variative (%)	*F_ST_* ^a^	*p*	*Nm* ^b^
Among populations	10	34.21	3.42	4.0%	0.043	0.042	5.639
Within populations	203	377.28	1.86	96%	…	…	…
Total	213	411.49	5.28	100%	…	…	…

^a^ Fixation index: *F_ST_* < 0.05 = low genetic differentiation. ^b^ *Nm* = absolute number of migrants per generation: *Nm* > 1 = great gene flow.

## Data Availability

All relevant data are within the paper.
